# Supplementary material for: Grid Cell Firing Patterns Maintain their Hexagonal Firing Patterns on a Circular Track
Source: Res Sq. 2023 Sep 19:rs.3.rs-3353284. Preprint. [Version 1] doi: 10.21203/rs.3.rs-3353284/v1 (PMC10543478; doi:10.21203/rs.3.rs-3353284/v1)
Supplement: Supplement 1 [file NIHPPRS3353284V1-supplement-1.pdf]

Supplementary Figures

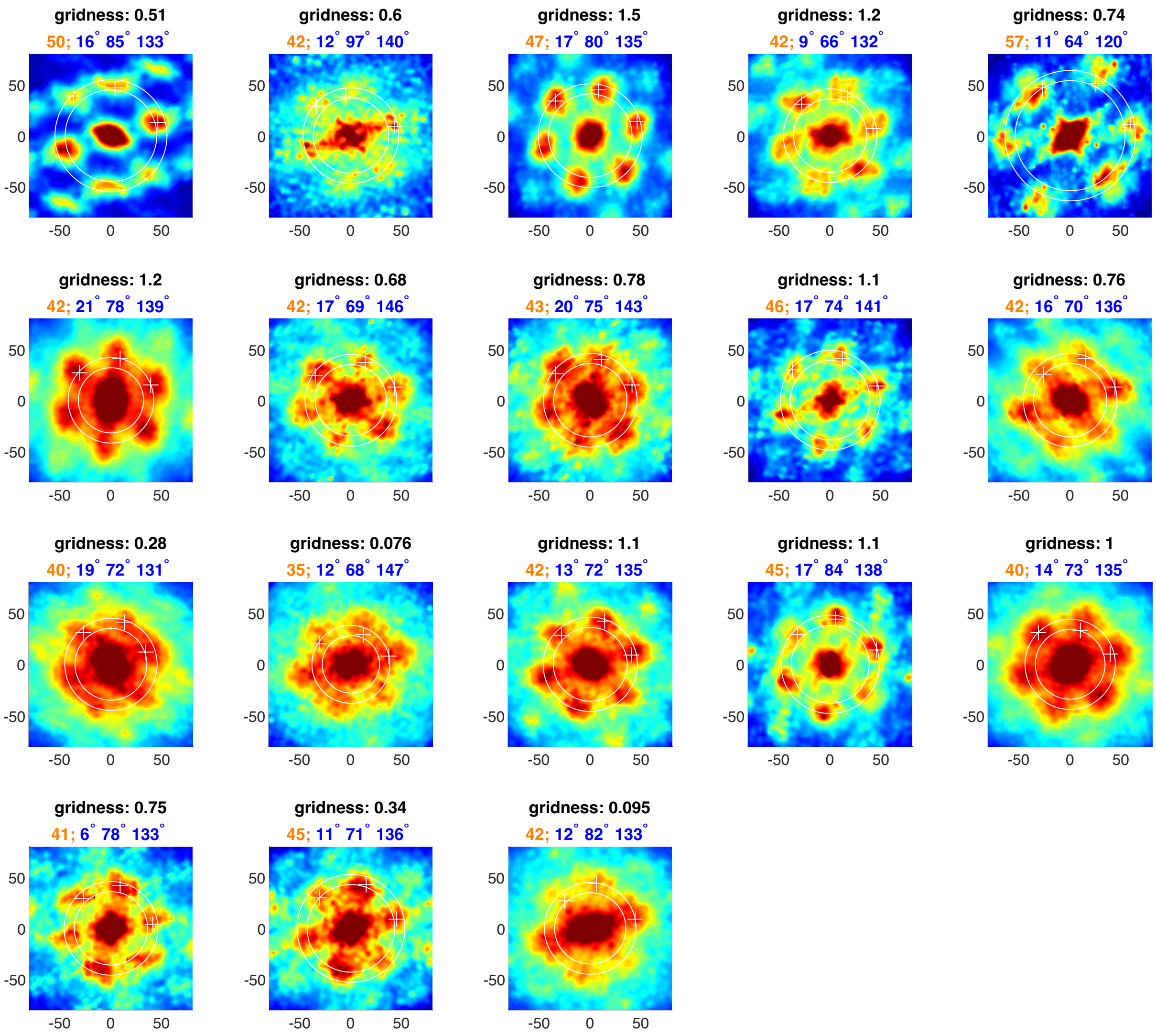

Figure S1. Autocorrelations of all 18 grid cells in Rat 1 in the open arena (Environment E_1). The inferred spacing and orientations are labeled with white circle and white pluses, respectively. Those values (black), together with the gridness score (blue), are shown for each cells.

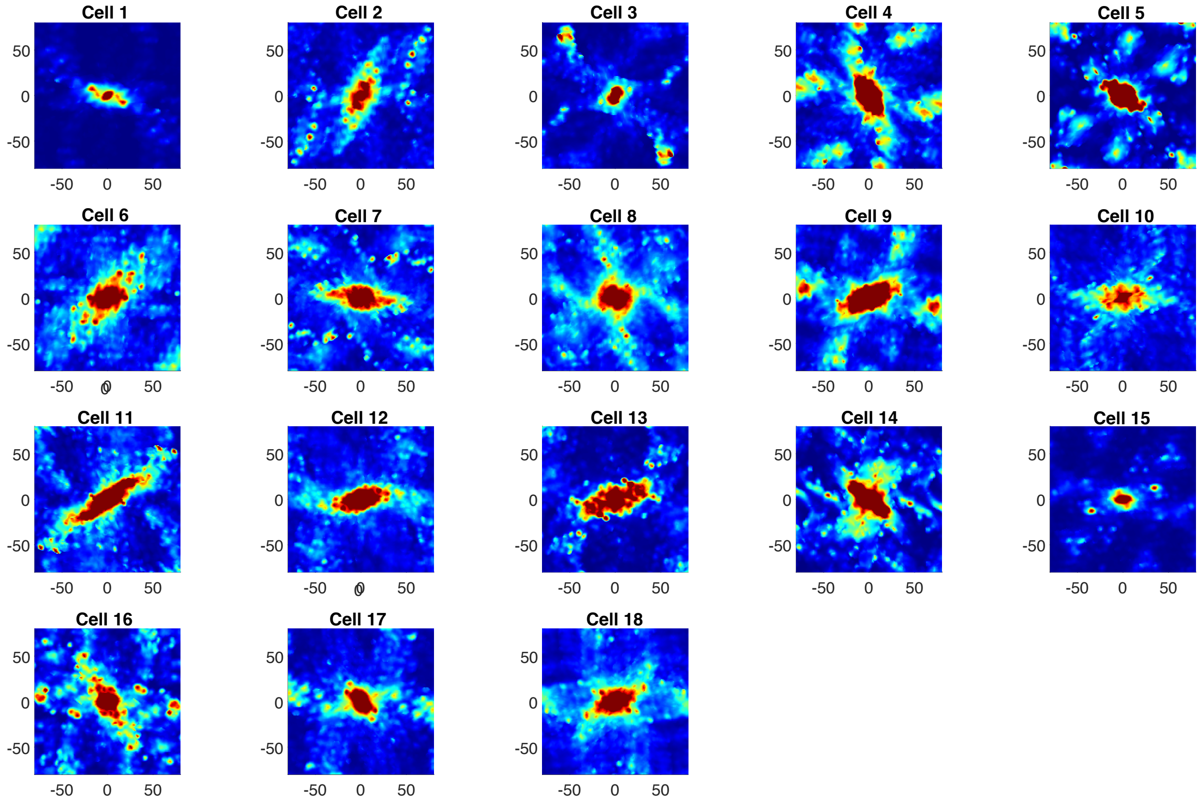

Figure S2. Autocorrelations of all 18 grid cells in Rat 1 on the circular track in the light condition (Environment E_2). A few cells have peaks that could be part of an underlying hexagonal lattice (e.g. Cells 5, 9, 15 and 17). However, the spatial structure of even these cells is unclear, and overall the individual autocorrelations do not clearly indicate a particular consistent spatial structure in the grid cell firing fields.

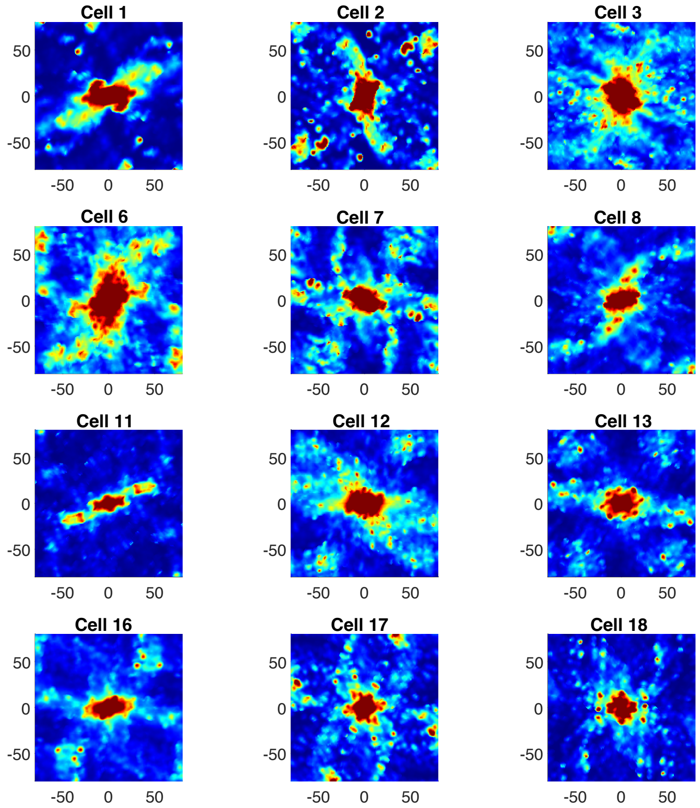

Figure S3. Autocorrelations of all 18 grid cells in Rat 1 on the circular track in the dark condition (Environment E_3). Like in E_1, a few cells have peaks that could be part of an underlying lattice (e.g. Cells 13-16). However, the overall spatial structure is again unclear from these individual autocorrelations.

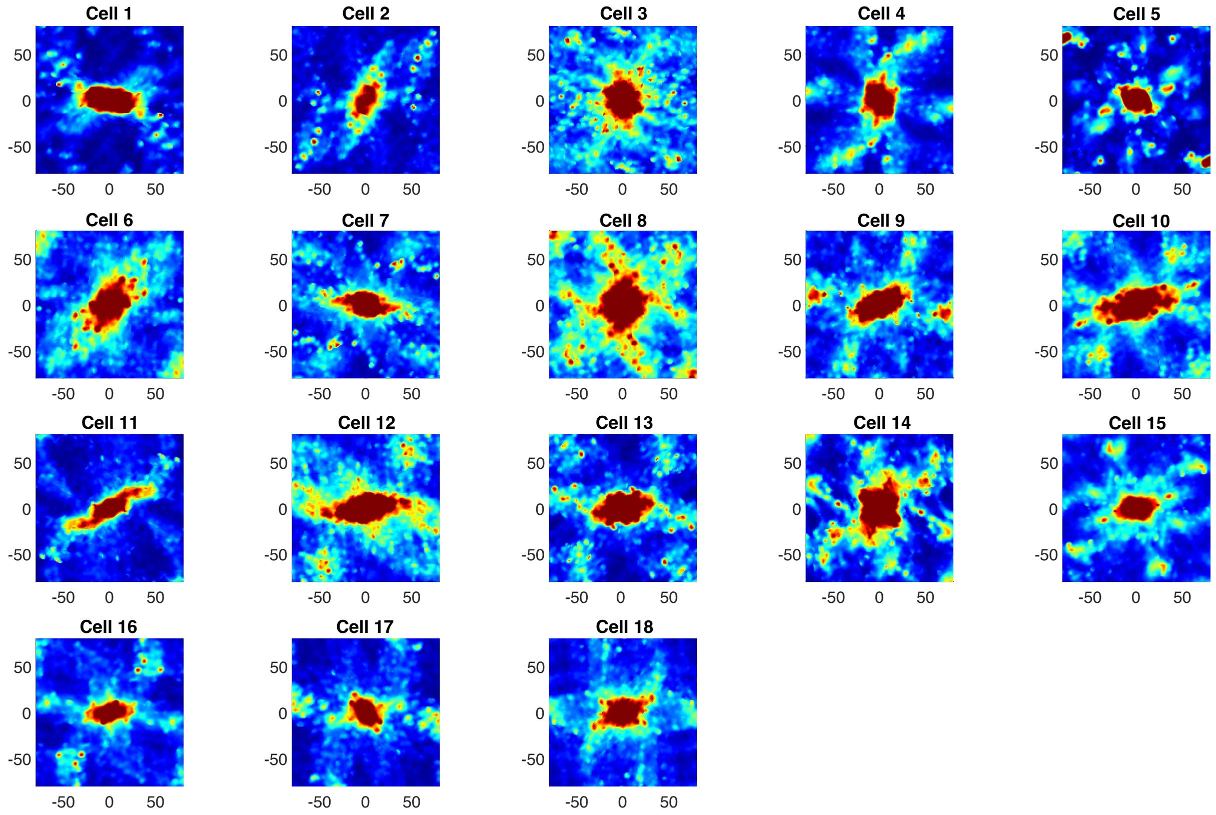

Figure S4. Combined autocorrelations of all 18 grid cells in Rat 1 on the circular track in both conditions. The combined autocorrelation for each grid cell is the sum of the autocorrelations in E_1 and E_2. While there is still no consistent spatial structure, a few cells show the ring of 6 evenly spaced peaks characteristic of a hexagonal lattice. Note that the autocorrelation of Cell 8 resembles the hexagonal structure, while only two peaks appear in E_2 and four in E_3.

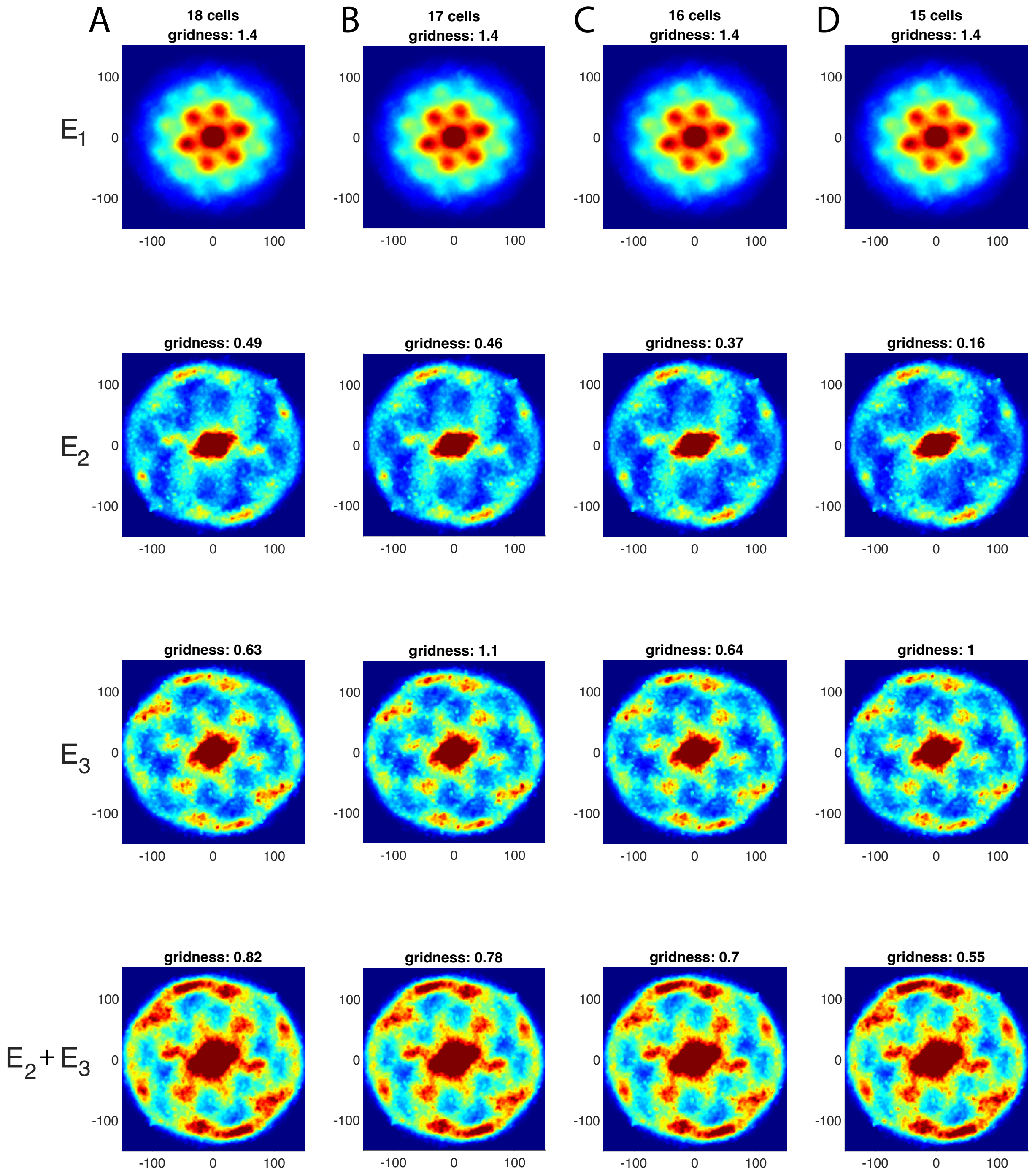

Figure S5. Robustness of results in Rat 1 to removing structured grid cells. To verify that the hexagonal structure in the population autocorrelation is a population effect that is not due to a few dominant grid cells, we recomputed the population autocorrelation while leaving out the grid cells with the clearest hexagonal structure in their individual autocorrelations in the combined environments (E_2+E_3, shown Supp. Fig. S4). (A) Population autocorrelation using all 18 grid cells. (B-D) Population autocorrelation using all cells except Cell 14 (B), Cells 14 and 8 (C), and Cells 14, 8 and 16 (D). The hexagonal structure is robust to removing these cells, particularly in Environments E_2 and $E_2 + E_3$.

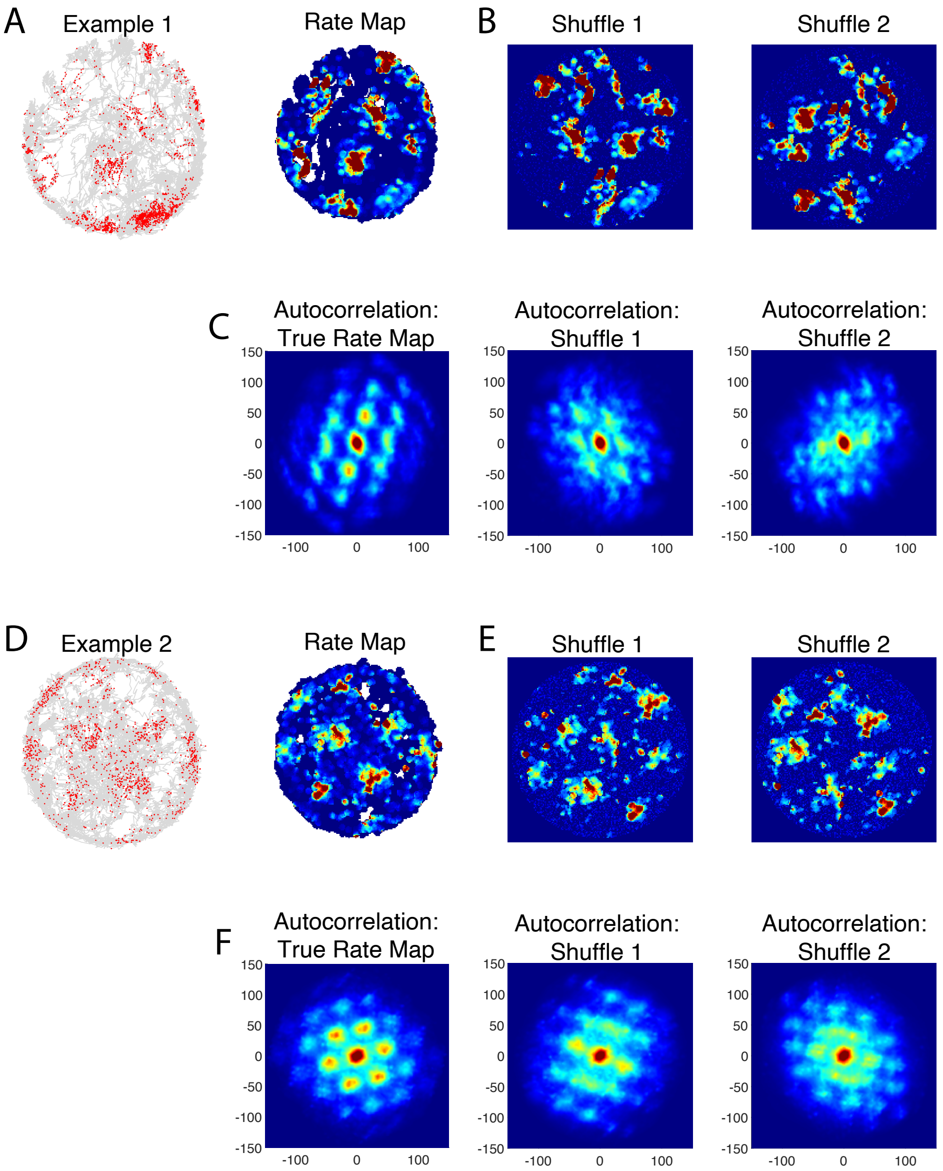

Figure S6. Examples of shuffle controls in the open arena (Environment E_1). (A) Data from an example cell in Rat 1. On the left, each red dot shows the location of the rat when this grid cell spiked, and the gray shows the rat's trajectory. The corresponding rate map is shown on the right. (B) Two example shuffles of the firing fields in the rate map. Note that the firing fields are shuffled, not the individual spikes. Thus the non-spatial firing rate statistics are preserved in the shuffles. (C) Autocorrelations of the true rate map, Shuffle 1 and Shuffle 2. (D-F) Same as A-C, but for a second example cell. As expected, the hexagonal pattern is much clearer in the autocorrelation of the true rate map than for the shuffles.

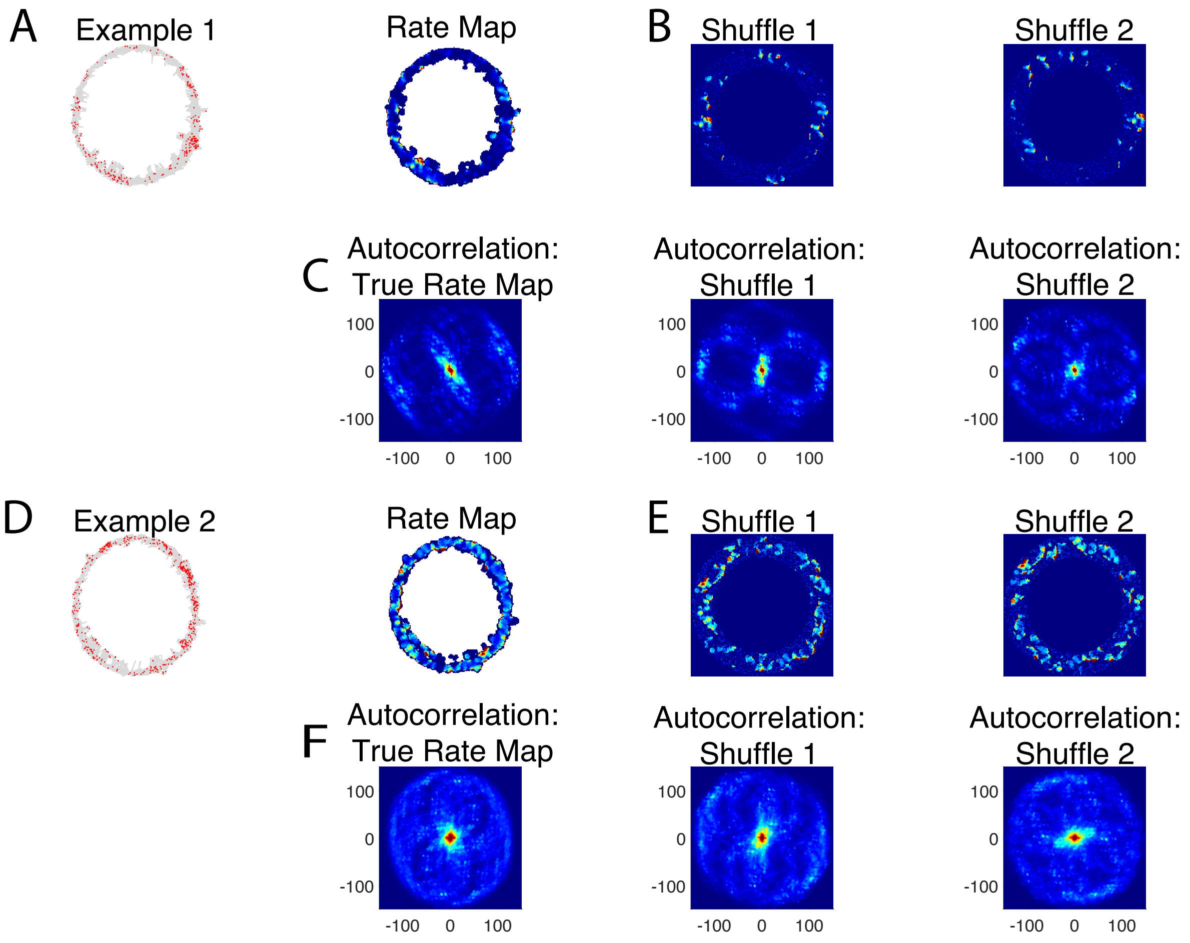

Figure S7. Examples of shuffle controls on the circular track (Environment E_3). (A) Data from an example cell in Rat 1. On the left, each red dot shows the location of the rat when this grid cell spiked, and the gray shows the rat's trajectory. The corresponding rate map is shown on the right. (B) Two examples shuffles of the firing fields in the rate map. As done for the open field environment, firing fields are kept intact so that the non-spatial statistics are preserved in the shuffles. (C) Autocorrelations of the true rate map, Shuffle 1 and Shuffle 2. (D-F) Same as A-C, but for a second example cell. The autocorrelations of individual cells are usually not informative about the underlying spatial firing pattern due to the sparsity of the data on the circular track.

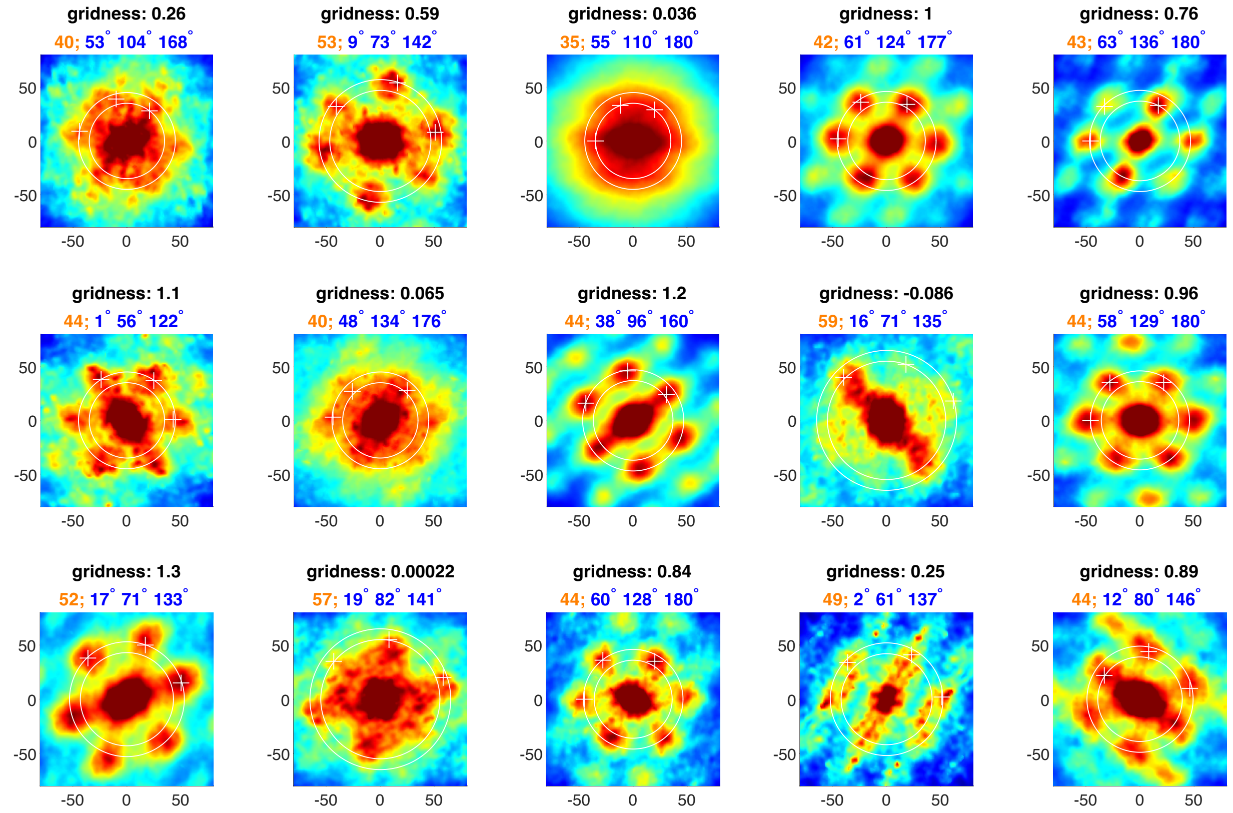

Figure S8. Autocorrelations of all 15 grid cells in Rat 2 in 2D arena.

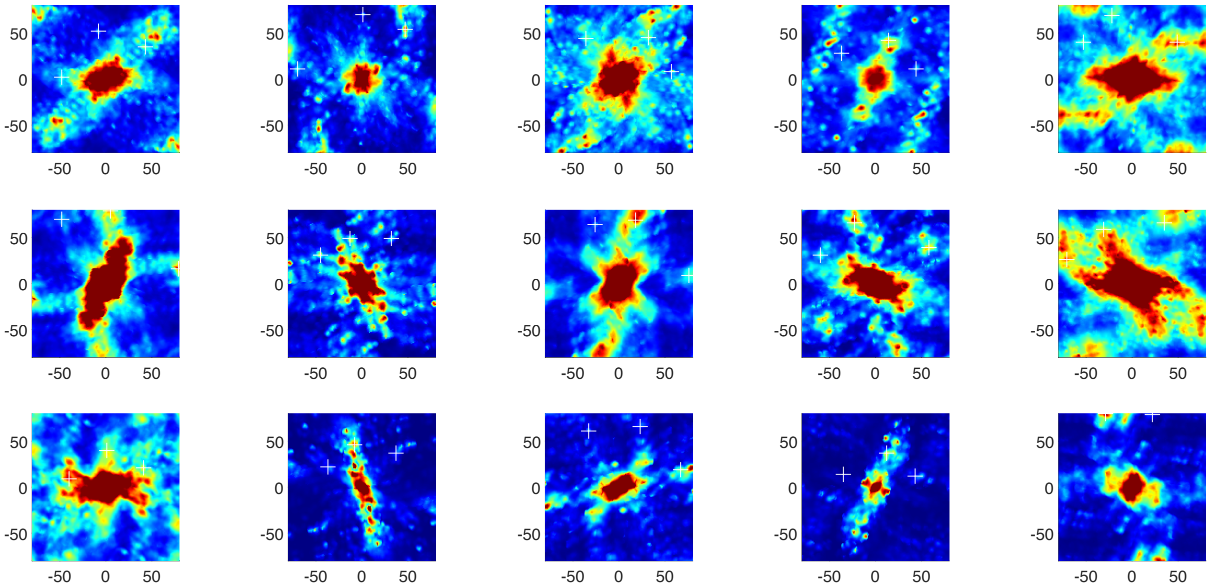

Figure S9. Autocorrelations of all 15 grid cells in Rat 2 on the light annlus.

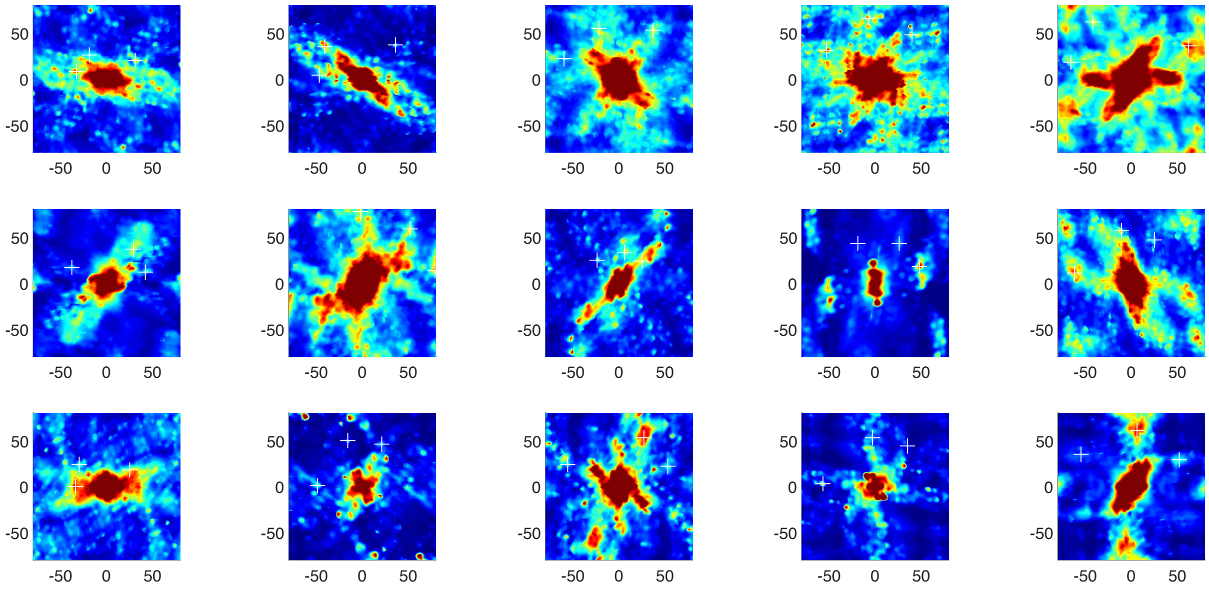

Figure S10. Autocorrelations of all 15 grid cells in Rat 2 on the dark annulus.

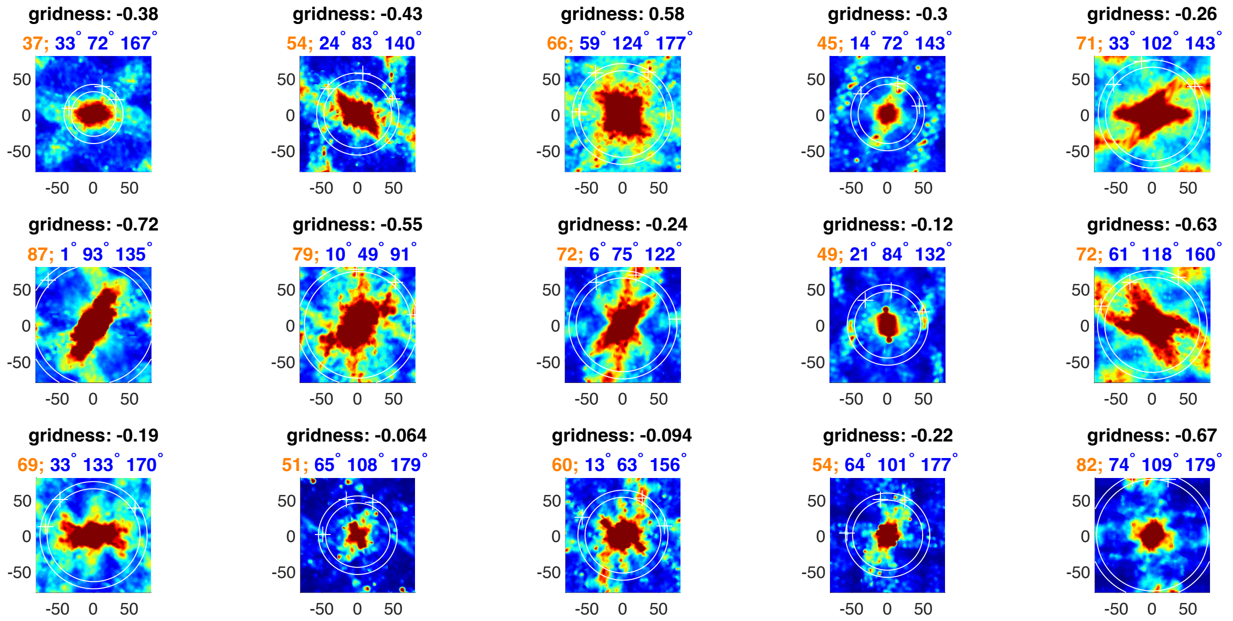

Figure S11. Autocorrelations of all 15 grid cells in Rat 2 on the light and dark annuli combined.
